# Evaluation of staff knowledge; attitudes and experience of breastfeeding on a mother and baby unit

**DOI:** 10.1192/bjo.2021.481

**Published:** 2021-06-18

**Authors:** Patrick Britto, Chidi Nwosu, Kate Seagar, Sarah Reed

**Affiliations:** 1 King's college london; 2 Kent and Medway NHS and Social Care Partnership Trust

## Abstract

**Aims:**

To evaluate the knowledge and experience of breastfeeding of staff working on a Mother and Baby Unit (MBU).

To assess the level of breastfeeding education of Staff on the MBU.

To identify any area of concern around breastfeeding on the MBU.

**Method:**

A fourteen question questionnaire was designed with assistance of the medical team, midwife, and health visitor on the unit. The questionnaire was comprised of questions requiring “yes/no” and free text responses alongside Likert scales. The questionnaire focused on staff experience on breastfeeding, education levels and whether they felt Mothers were sufficiently supported. This questionnaire was distributed to all staff groups within the team to ascertain the level of expertise. 29 questionnaires were returned from a staff team of 31.

**Result:**

Staff on this unit is made up of Multi-disciplinary professionals. Most respondents were Nursery Nurses (15%). 79% of staff had a lived experience of breastfeeding. Only 5 out of 29 respondents have had any breastfeeding training which was mainly in-house training, and these were the Health Visitor; Midwife and Nursery nurses. Of the respondents, 21% felt mothers who choose breastfeeding as their preferred mode of infant feeding were not adequately supported on the MBU. Seven percent were unsure and 72% felt women were adequately supported. 54% of staff were not aware of breastfeeding initiatives. 63% were able to list contraindications including names of psychotropic medications as well as personal choice and past medical history. The median rating in relation to confidence in skills on Likert scale of 1-10 was 5.

**Conclusion:**

23 out of 29 professionals felt that Training would increase their confidence and skills in breast feeding support for women admitted to the unit. There is clear indication from the Staff Members that mothers on the MBU who choose breastfeeding as their preferred mode of infant feeding require further support. There is lack of confidence in staff's breastfeeding support in the MBU. An evaluation of patient's own experience of breastfeeding support they receive from staff is being undertaken alongside this, but data will be analysed later.

